# Prognostic analysis of three forms of Ki‐67 in patients with breast cancer with non‐pathological complete response before and after neoadjuvant systemic treatment

**DOI:** 10.1002/cam4.5693

**Published:** 2023-02-16

**Authors:** Weiwei Zhang, Yinggang Xu, Ye Wang, Jinzhi He, Rui Chen, Xinyu Wan, Wenjie Shi, Xiaofeng Huang, Xiaoqing Shi, Jue Wang, Xiaoming Zha

**Affiliations:** ^1^ Department of Breast Disease The First Affiliated Hospital of Nanjing Medical University Nanjing China; ^2^ Collaborative Innovation Center for Cancer Personalized Medicine Nanjing Medical University Nanjing China

**Keywords:** breast cancer, cell cycle, neoadjuvant chemotherapy, prognosis

## Abstract

**Background:**

Patients who do not achieve a pathological complete response (pCR) after neoadjuvant systemic treatment (NST) have a significantly worse prognosis. A reliable predictor of prognosis is required to further subdivide non‐pCR patients. To date, the prognostic role in terms of disease‐free survival (DFS) between the terminal index of Ki‐67 after surgery (Ki‐67_T_) and the combination of the baseline Ki‐67 at biopsy before NST (Ki‐67_B_) and the percentage change in Ki‐67 before and after NST (Ki‐67_C_) has not been compared.

**Aim:**

This study aimed to explore the most useful form or combination of Ki‐67 that can provide prognostic information to non‐pCR patients.

**Patients and Methods:**

We retrospectively reviewed 499 patients who were diagnosed with inoperable breast cancer between August 2013 and December 2020 and received NST with anthracycline plus taxane.

**Results:**

Among all the patients, 335 did not achieve pCR (with a follow‐up period of ≥1 year). The median follow‐up duration was 36 months. The optimal cutoff value of Ki‐67_C_ to predict a DFS was 30%. A significantly worse DFS was observed in patients with a low Ki‐67_C_ (*p* < 0.001). In addition, the exploratory subgroup analysis showed relatively good internal consistency. Ki‐67_C_ and Ki‐67_T_ were considered as independent risk factors for DFS (both *p* < 0.001). The forecasting model combining Ki‐67_B_ and Ki‐67_C_ showed a significantly higher area under the curve at years 3 and 5 than Ki‐67_T_ (*p* = 0.029 and *p* = 0.022, respectively).

**Conclusions:**

Ki‐67_C_ and Ki‐67_T_ were good independent predictors of DFS, whereas Ki‐67_B_ was a slightly inferior predictor. The combination of Ki‐67_B_ and Ki‐67_C_ is superior to Ki‐67_T_ for predicting DFS, especially at longer follow‐ups. Regarding clinical application, this combination could be used as a novel indicator for predicting DFS to more clearly identify high‐risk patients.

## Graphical abstract

Patients who do not achieve a pathological complete response (pCR) after neoadjuvant systemic treatment (NST) have a significantly worse prognosis. This study aimed to explore the most useful form or combination of Ki‐67 including the baseline Ki‐67 at biopsy before NST (Ki‐67_B_), the percentage change in Ki‐67 before and after NST (Ki‐67_C_), and the terminal index of Ki‐67 after surgery (Ki‐67_T_), that can provide prognostic information to non‐pCR patients. We retrospectively reviewed 499 patients, and 388 patients did not achieve pCR. The forecasting model combining Ki‐67_B_ and Ki‐67_C_ showed a significantly higher area under the curve (AUC) at years 3 and 5 than Ki‐67_T_ (*p* = 0.029 and *p* = 0.022, respectively). The combination of Ki‐67_B_ and Ki‐67_C_ is superior to Ki‐67_T_ for predicting DFS, especially at longer follow‐ups.

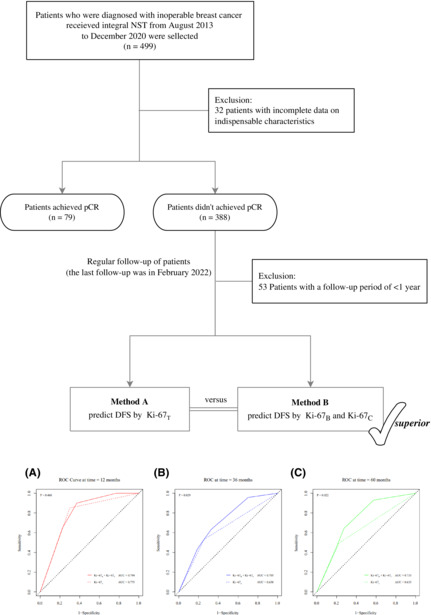

## 1 INTRODUCTION

Neoadjuvant systemic treatment (NST) is becoming increasingly popular for the treatment of breast cancer because of its superiority in evaluating drug susceptibility and creating opportunities for operation or even breast‐conserving surgery.^1^, ^2^ Achievement of pathological complete response (pCR) after NST is a good surrogate endpoint for better survival.^3^, ^4^ However, patients who achieve pCR after NST remain a minority, and more than half still have cancer in the breast or axillary lymph nodes (ALNs) after NST.^5^ Interestingly, survival among these patients varies widely.^6^ Thus, the identification of prognostic indicators is crucial for this patient population. Consequently, much effort is being invested in searching for a reliable predictor of prognosis to further subdivide non‐pCR patients.^7^

Ki‐67 is a non‐histone nuclear protein in proliferating cells that is present in all phases of the cell cycle, except for G0.^8^ It is an important indicator of the degree of tumor malignancy and is used to classify breast cancer, predict the chemosensitivity of tumors, and evaluate prognoses.^9^, ^10^, ^11^ For the non‐pCR patient population, three factors related to Ki‐67 can be determined: the baseline Ki‐67 at biopsy before NST (Ki‐67_B_), the percentage change in Ki‐67 before and after NST (Ki‐67_C_), and the terminal index of Ki‐67 after surgery (Ki‐67_T_).

Ki‐67_B_ has been reported to predict the efficacy of NST.^12^, ^13^ Patients whose Ki‐67_B_ levels are initially high are more sensitive to chemotherapy and are more likely to achieve pCR.^12^ However, the role of Ki‐67_B_ in predicting the prognosis remains controversial.^14^, ^15^, ^16^ In contrast, the ability of Ki‐67_C_ or Ki‐67_T_ to predict survival is relatively clear, and patients with a low level of Ki‐67_T_ or high Ki‐67_C_ are less likely to develop recurrence and metastasis.^11^, ^15^, ^17^

Theoretically, Ki‐67_T_ reflects terminal proliferation of tumors after receiving NST.^18^ This value is complicated because it is affected by several factors. Unlike Ki‐67_T_, Ki‐67_B_ reflects the baseline proliferation of the tumor tissue before receiving any treatment, and Ki‐67_C_ reflects the sensitivity of the tumor to NST, together with therapeutic efficacy.^19^ Thus, the malignancy of the tumor itself and the treatment efficacy can be analyzed using Ki‐67_B_ and Ki‐67_C_, respectively. However, no studies have discussed whether Ki‐67_B_ and Ki‐67_C_ can predict the prognosis of patients more accurately than Ki‐67_T_.

To date, the prognostic role in terms of disease‐free survival (DFS) between Ki‐67_T_ and the combination of Ki‐67_B_ and Ki‐67_C_ have not been systematically compared. Thus, we designed this study to search for an appropriate cutoff value for Ki‐67_C_ and to explore the most useful combination form of Ki‐67 that can provide prognostic information to patients who have completed NST but do not achieve pCR.

## 2 MATERIALS AND METHODS

### 2.1 Patients

We retrospectively reviewed the medical records of 499 patients who were diagnosed with inoperable breast cancer and received an NST with anthracycline plus taxane at the First Affiliated Hospital of Nanjing Medical University (Nanjing, China) between August 2013 and December 2020. We excluded patients with a history of malignancy or chemotherapy, bilateral breast cancer, incomplete NST, distant metastasis found before or during NST, unavailable information regarding tumor characteristics, and follow‐up of <1 year.

This study was conducted in accordance with the Declaration of Helsinki of the World Medical Association (revised 2013).

## 3 METHODS

All breast cancer cases were diagnosed using core needle biopsy. Then, the specimen was further examined using immunohistochemistry (IHC) and considered hormone receptor (HR)‐positive if >1% of cells showed the expression of estrogen receptors or progesterone receptors. In addition, it was defined as HER2‐positive if in situ hybridization revealed HER2 gene amplification or IHC staining of HER2 was 3+.

Breast cancer cases were further differentiated into four molecular subtypes according to their HR and HER status: HR+/HER2−, HR+/HER2+, HR−/HER2+, or HR−/HER2− (triple‐negative, TN). Clinical tumor (cT) size and clinical lymph node (cN) classification were determined using the TNM Staging System for Breast Cancer (8th edition), as per the American Joint Committee on Cancer.

All patients received chemotherapy regimens of four cycles of epirubicin/cyclophosphamide followed by four cycles of paclitaxel or docetaxel. HER2‐positive patients received targeted therapy with trastuzumab ± pertuzumab, beginning concurrently with paclitaxel or docetaxel. After completing the systemic NST, patients were scheduled for surgery within 1 month. The decision to perform mastectomy or breast‐conserving surgery was made according to the tumor burden of the breast and the patient's willingness. ALN dissection was performed in patients considered to have ALN metastasis before undergoing NST. Otherwise, a sentinel lymph node biopsy was performed before the NST. All the surgically resected specimens were sent to an experienced pathologist for further examination. A pCR was defined as the absence of invasive cancer in the breast as well as ALNs, regardless of the presence of ductal carcinoma in situ of the breast.^3^ Patients were then grouped according to whether pCR was achieved postoperatively.

All specimens were tested for Ki‐67 (clone MX006, MAB‐0672; Fuzhou Maixin Biotech Co., Ltd.) before and after undergoing the NST. The percentage of positively stained cells, defined as any degree of brown staining in the nucleus, is reported. According to the Guidelines of the Chinese Society of Clinical Oncology (CSCO) and the 2021 St. Gallen expert Consensus, a Ki‐67_B_ or Ki‐67_T_ index >30% was considered high, and an index <30% was considered low.^20^ According to the analysis of the receiver operating characteristic (ROC) curve, an increased or ≤ 30% reduction in Ki‐67_C_ was considered a low Ki‐67_C_, and a reduction >30% was considered a high Ki‐67_C_. All pathologies identified using core needle biopsy before NST and the pathological responses of the primary site and ALN after NST were assessed microscopically by a specialist pathologist and reviewed by another professional pathologist.

In the postoperative intensive adjuvant phase of non‐pCR patients, those with HER2+ disease were proposed to receive dual‐targeted therapy or T‐DM1 for 1 year.^21^ HR+ patients were proposed to receive adjuvant endocrine therapy (aromatase inhibitor or tamoxifen with or without ovarian function suppression) for at least 5 years.^22^, ^23^ Patients with TN have been proposed to receive orally administered capecitabine for 1 year.^24^ The decision to administer radiotherapy at a specific site was made by a radiologist.

Our study protocol included regularly scheduled follow‐ups, and the last follow‐up was conducted in February 2022. DFS was considered as the primary outcome measure. It was defined as the interval between the time of surgery and tumor recurrence, metastasis, tumor‐related disease, or the date of censoring. Patients with a follow‐up period of <1 year were excluded.

### 3.1 Statistical analyses

The probability of DFS was estimated using the Kaplan–Meier method and the log‐rank test. We used univariate and multivariate Cox regression models to analyze the associations of DFS with the listed variables and the likelihood ratio test to establish the multivariate Cox model. In addition, a time‐dependent ROC curve was drawn at years 1, 3, and 5 to compare the sensitivity and specificity of the prognostic ability between different Ki‐67 categories. The area under the curve (AUC) was calculated using the ROC curve. The Youden J statistic was used to confirm the optimal cutoff value. Statistical significance was set at *P* < 0.05. All statistical analyses were performed using IBM SPSS Statistics v26.0 for Windows (IBM Corporation, NY, USA) and R v4.1.3 software (the R Foundation for Statistical Computing, Vienna, Austria).

## 4 RESULTS

### 4.1 Patient and tumor baseline characteristics among non‐pCR patients

**FIGURE 1:**
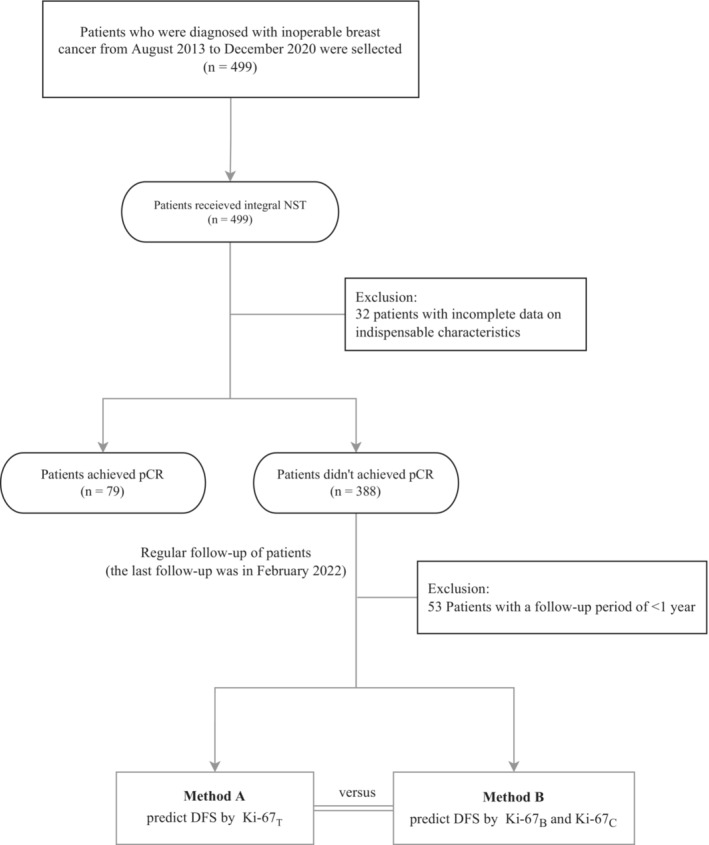
Study design and patient flow. _B_, baseline; _C_, percentage change; DFS, disease‐free survival; NST, neoadjuvant systemic treatment; pCR, pathological complete response; _T_, terminal.

We screened 499 patients who underwent an integral NST (Figure 1). Of these, 32 were excluded because of incomplete data on indispensable characteristics. Postoperative analysis of surgically excised specimens revealed that pCR was not achieved in 388 patients. Furthermore, 53 patients were excluded because their follow‐up period was <1 year. Finally, we included a total of 335 patients who had not yet achieved pCR after NST.

The patient and tumor characteristics of the 335 patients are presented in Table 1, of which 41 (12.2%) were aged ≤35 years. HR+/HER2− patients accounted for 54.6% of the population; the proportions of HR+/HER2+ and TN patients were similar (16.4% and 16.1%, respectively), and HR−/HER2+ patients made up the smallest proportion (12.8%). Clinical assessment revealed that 97.9% of patients were classified as cT2 or higher, 88.1% were considered ALN metastatic, and 53.1% were classified as cN2. Most patients had TNM stage IIIA disease. Only 6.6% (22/335) of patients opted for breast‐conserving surgery. Pathologically, the majority of surgically excised specimens were diagnosed as ductal carcinomas (30/335, 91.6%). There were 213 (63.6%) patients with Ki‐67_B_ > 30%. Only 32.5% (109/335) of patients had a high level of Ki‐67_T_. There were 133 (39.7%) patients with low Ki‐67_C_ (increased or ≤ 30% reduction) (Table 1). The optimal cutoff value of Ki‐67_C_ to predict a DFS was 30% (Figure SS1).

**TABLE 1:** Patient and tumor characteristics of non‐pCR patients.

Characteristics	Non‐pCR patients (*n* = 335)	
	No.	%
Age at diagnosis (years)		
≤35	41	12.2
>35	294	87.8
Subtype		
HR+/HER2−	183	54.6
HR+/HER2+	55	16.4
HR−/HER2+	43	12.8
TN	54	16.1
Clinical tumor size		
cT1	7	2.1
cT2	209	62.4
cT3	65	19.4
cT4	54	16.1
Clinical node stage		
cN0	40	11.9
cN1	65	19.4
cN2	178	53.1
cN3	52	15.5
TNM stage		
IIA	33	9.9
IIB	51	15.2
IIIA	162	48.4
IIIB	37	11.0
IIIC	52	15.5
Surgery		
Mastectomy surgery	313	93.4
Breast‐conserving surgery	22	6.6
Postoperative pathology		
Ductal	307	91.6
Lobular	10	3.0
Other	4	1.2
Carcinoma only in ALN	14	4.2
Ki‐67_B_ index		
≤30%	122	36.4
>30%	213	63.6
Ki‐67_C_ index		
Increased or ≤ 30% reduction	133	39.7
>30% reduction	202	60.3
Ki‐67_T_ index		
≤30%	226	67.5
>30%	109	32.5

Abbreviations: ALN, axillary lymph node; _B_, baseline; _C_, percentage change; cN, clinical node; cT, clinical tumor; HR, hormone receptor; non‐pCR, not achieved pathological complete response; _T_, terminal; TN, triple‐negative (HR−/HER2−).

### 4.2 Subgroup analysis of DFS according to Ki‐67_C_ level

**FIGURE 2:**
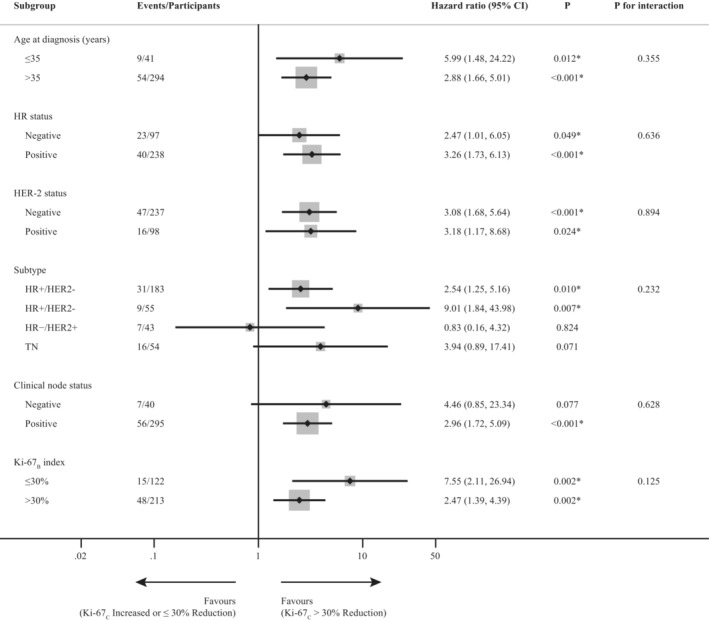
Subgroup analysis of disease‐free survival related to Ki‐67_C_ among non‐pCR patients. _B_, baseline; _C_, percentage change; CI, confidence interval; HR, hormone receptor; Non‐pCR, not achieved pathological complete response; TN, triple‐negative (HR−/HER2−).

A significantly worse DFS (hazard ratio 3.10, 95% confidence interval [CI]: 1.86–5.19; *p* < 0.001) was observed in patients with low Ki‐67_C_ (Table 2). In the subgroup analysis of age at diagnosis, HR status, HER‐2 status, and Ki‐67_B_ level, the results for each subgroup were similar; patients with a high level of Ki‐67_C_ were correlated with better DFS (Figure 2). In contrast, the reduction in Ki‐67_C_ only played a satisfactory prognostic role in DFS in the HR+/HER2− and HR+/HER2+ subtypes and in the cN‐positive subgroup. There was no significant interaction between every subgroup and the level of Ki‐67_C_ (all *p* > 0.05).

**TABLE 2:** Univariate Cox regression analysis of DFS among non‐pCR patients.

Variables	Unadjusted hazard ratio	95% CI	*p*‐value
Age at diagnosis >35 years	0.68	0.34–1.38	0.285
HR positive	0.66	0.40–1.11	0.114
HER‐2 positive	0.79	0.45–1.39	0.415
Clinical tumor size (ordinal, range 1–4)	1.23	0.91–1.66	0.173
Clinical node stage (ordinal, range 0–3)	1.87	1.35–2.58	<0.001*
Subtype			0.089
HR+/HER2−	1	Ref.	
HR+/HER2+	0.98	0.47–2.06	0.958
HR−/HER2+	0.93	0.41–2.12	0.866
TN	2.07	1.13–3.79	0.019*
Breast‐conserving surgery	0.87	0.32–2.40	0.792
Ki‐67_B_ > 30%	2.12	1.19–3.79	0.011*
Ki‐67_C_ increased or ≤ 30% reduction	3.10	1.86–5.19	<0.001*
Ki‐67_T_ > 30%	3.04	1.85–5.00	<0.001*

Abbreviations: _B_, baseline; _C_, percentage change; CI, confidence interval; DFS, Disease‐free survival; HR, hormone receptor; non‐pCR, not achieved pathological complete response; _T_, terminal; TN, triple‐negative (HR−/HER2−).

* *p*‐value <0.05.

### 4.3 Univariate and multivariate analyses of DFS among non‐pCR patients

**FIGURE 3:**
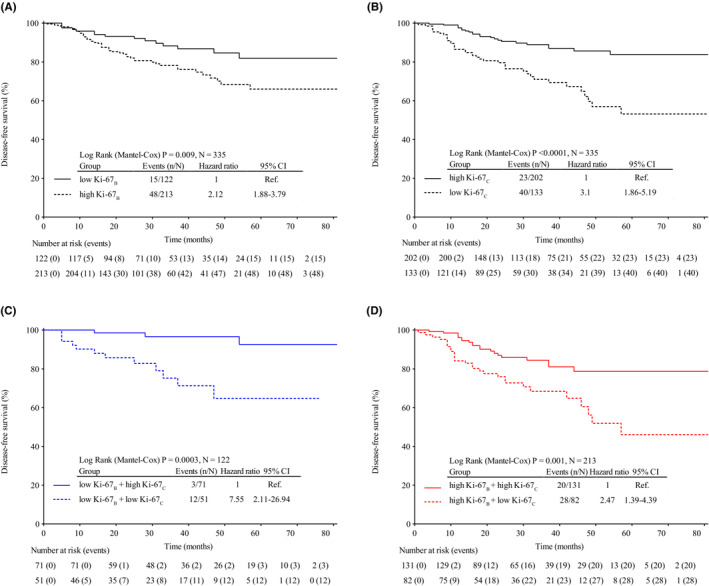
Kaplan–Meier survival curve for disease‐free survival by Ki67_B_ (A), Ki67_C_ (B), and combination of Ki‐67_B_ and K‐67_C_ (C, D). _B_, baseline; _C_, percentage change; CI, confidence interval; low Ki‐67_B_ = Ki‐67_B_ ≤ 30%; high Ki‐67_B_ = Ki‐67_B_ > 30%; low Ki‐67_C_ = Ki‐67_C_ increased or ≤ 30% reduction; high Ki‐67_C_ = Ki‐67_C_ > 30% reduction.

The median follow‐up period of the non‐pCR patients was 36 months (range, 12–98 months). Univariate Cox regression analysis of non‐pCR patients revealed that cN, subtype, Ki‐67_B_, Ki‐67_C_, and Ki‐67_T_ were significantly associated with DFS (Table 2). Figure 3 shows the Kaplan–Meier plots for DFS grouped by Ki67_B_ and Ki67_C_.

We used two methods to build regression models (Figure 1 and Table 3) to compare the difference in the prediction of prognosis between Ki‐67_T_ and the combination of Ki‐67_B_ and Ki‐67_C_. In method A, cN stage and Ki‐67_T_ were considered independent risk factors for DFS. Patients in the high Ki‐67_T_ group had worse DFS than those in the low Ki‐67_T_ group (adjusted hazard ratio: 2.77, CI: 1.68–4.56, *p* < 0.001) (Table 3). In method B, cN and Ki‐67_C_ were also considered as independent risk factors for DFS. The low Ki‐67_C_ group was more prone to recurrence and metastasis than the high Ki‐67_C_ group (adjusted hazard ratio: 2.90, CI: 1.73–4.86, *p* < 0.001) (Table 3). Furthermore, we used the Kaplan–Meier method to plot DFS curves for patients in the Ki‐67_B_ subgroup (Figure 3). In the low Ki‐67_B_ subgroup, low Ki‐67_C_ was associated with worse DFS (hazard ratio: 7.55, CI: 2.11–26.94). In the high Ki67_B_ subgroup, the results were similar to those mentioned above (hazard ratio: 2.47, CI: 1.39–4.39).

**TABLE 3:** Multivariate Cox regression analysis of DFS among non‐pCR patients using two methods.

Variables	Adjusted hazard ratio	95% CI	*p*‐value
Method A			
Clinical node stage (ordinal, range 0–3)	1.74	1.27–2.41	0.001*
Ki‐67_T_ > 30%	2.77	1.68–4.56	<0.001*
Method B			
Clinical node stage (ordinal, range 0–3)	1.64	1.19–2.26	0.002*
Ki‐67_B_ > 30%	1.75	0.97–3.17	0.064
Ki‐67_C_ increased or ≤ 30% reduction	2.90	1.73–4.86	<0.001*

Abbreviations: _B_, baseline; _C_, percentage change; CI, confidence interval; DFS, disease‐free survival; non‐pCR, not achieved pathological complete response; _T_, terminal.

* *p*‐value <0.05.

### 4.4 Time‐dependent ROC curve analysis

**FIGURE 4:**
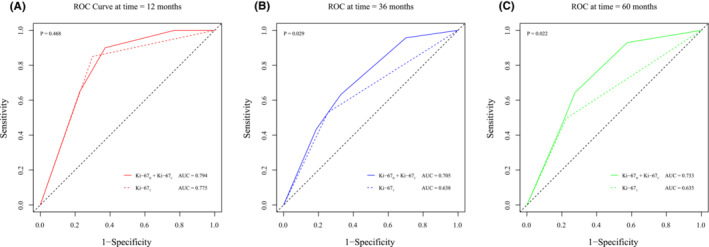
Time‐dependent ROC curve analysis for the Cox regression models at year 1 (A), 3 (B), and 5 (B). AUC, area under the curve; _B_, baseline; _C_, percentage change; ROC, receiver operating characteristic; _T_, terminal.

A time‐dependent ROC curve was drawn according to the Cox regression model established by Ki‐67_T_ and the combination of Ki‐67_B_ and Ki‐67_C_ for years 1, 3, and 5 (Figure 4), and the AUC value was further estimated. There was a statistically significant difference in the AUC values at year 3 and year 5 between the two forecasting models (70.5% vs. 63.8%, *p* = 0.029 and 73.3% vs. 63.5%, *p* = 0.022, respectively).

## 5 DISCUSSION

This study found that, individually, both Ki‐67_C_ and Ki‐67_T_ were independent predictors of DFS, whereas Ki‐67_B_ was slightly inferior. Patients with high levels of Ki‐67_C_ had a better prognosis, and this result showed relatively good internal consistency in the subgroup analysis. In this study, the prognostic value of Ki‐67_C_ in the HR‐positive subgroup was better than that in the HR‐negative subgroup. Similarly, one clinical trial showed that the Ki‐67 index after 2 weeks of perioperative endocrine therapy among HR‐positive patients was a better prognostic indicator than Ki67_B_.^19^ Ki‐67_B_ had a certain effect on predicting prognosis in univariate Cox regression analysis in this study. However, after adjusting for other confounding factors (cN and Ki‐67_C_), the prediction of DFS using Ki‐67_B_ was considered unsatisfactory. These results were similar to those of other clinical studies.^11^, ^15^ So far, no other relevant study has discussed further subgroup analysis of Ki‐67_C_ according to Ki‐67_B_ in DFS among non‐pCR patient populations. In this study, despite of whether the patients were classified into the low or high Ki‐67_B_ subgroup, those who showed a high reduction in Ki‐67_C_ had better DFS than those who did not. These results suggest a relatively poor predictive ability for Ki‐67_B_. As the DFS of patients with low Ki‐67_B_ but low Ki‐67_C_ was not very satisfactory in this study, this patient population must not be neglected in clinical follow‐up and treatment. Subsequent adjuvant therapy for this patient group should be emphasized and studied further; for example, the use of CDK4/6 inhibitors in HR‐positive patients or antibody‐drug conjugates in HER2‐positive patients.^25^, ^26^

In addition, this study revealed that the combination of Ki‐67_C_ and Ki‐67_B_ is superior to Ki‐67_T_ in predicting DFS in patients with breast cancer. At present, most studies only consider the respective relationship between K‐67_B_, Ki67_C_, and Ki‐67_T_, but do not propose combining the first two to produce a new prediction indicator, as in this study.^12^, ^16^ In the DFS predictions at year 1, both groups had high AUCs of approximately 78%. Interestingly, analysis of the prediction effect of a longer follow‐up time (3 and 5 years) revealed that the Ki‐67_T_ has deficiencies than the combination of Ki‐67_B_ and Ki‐67_C_ in predicting the long‐term DFS. This indicates that the combination of Ki‐67_B_ and Ki‐67_C_ has advantages in predicting long‐term prognosis and should be given more attention by clinicians in the enhanced treatment in the future.

It is feasible to use 30% as the cutoff value for Ki‐67 (whether Ki67_B_, Ki67_T_, or Ki67_C_) to predict the prognosis among non‐pCR patients with breast cancer in this study. Since the determined values of Ki‐67_B_ and Ki‐67_T_ may differ among pathological experimental centers, a median cutoff value of 20%–30% or the median value across testing laboratories can be used as the cutoff value.^27^ A cutoff value of 30% was used in this study, according to the CSCO and 2021 St. Gallen expert consensus guidelines.^20^ In addition, the majority of studies focus on the absolute value of Ki‐67 changes in predicting prognosis, while few discuss the percentage change. It is worth noting that, we analyzed the percentage of change in Ki‐67 values instead of the commonly used absolute change.^28^, ^29^ The percentage change can better reflect the overall change in Ki‐67 and not just the stacking of absolute values. Because of the different detection methods and experimental centers, there are deviations in the measured absolute value of Ki‐67,^30^ and the introduction of the concept of percentage change can make it universal for clinical applications. Furthermore, it removes interference from the initial Ki‐67_B_ and allows the evaluation of drug susceptibility in a more independent and systematic manner. Since research on the definition of Ki‐67_C_ is scarce, we thereby determined the optimal cutoff value of 30% by performing a time‐dependent ROC curve analysis at year 3 and employing the Youden index. One study by Matsubara et al. also focused on the percentage change in Ki‐67 decline. The difference between their study and ours is that they divided the patients into three groups: high‐reduction, low‐reduction, and increase groups, so there were two cutoff values: one was 80% and another is 0%.^29^ However, in Matsubara's study, high reduction in Ki‐67 comprised only 14.6% of all non‐pCR patients. And most patients (63%) were concentrated in the low‐reduction group, resulting in no good discrimination for this group of the patients. In contrast, setting a 30% cutoff value in this study is more scientific and intuitive.

There are few things to explain here. More than half of the non‐pCR patient population (183/335) were HR+/HER2−. This might be because these molecularly subtyped patients were not sufficiently sensitive to NST.^5^ The proportion of HER2‐positive patients in this study was low (<30%). Owing to the accessibility of dual‐target therapy in China, only about 11.2% (11/98) of all HER2‐positive patients were treated with double‐target therapy in this study. Therefore, this issue has not been discussed separately here.

This study has several strengths. First, we propose a novel concept of combining Ki‐67_C_ and Ki‐67_B_ to predict prognosis, which has never been involved in previous studies. Second, we recruited only patients who did not achieve a pCR in this study. This is because non‐pCR patients have a significantly worse prognosis than those with pCR, and this specific patient group is the focus of our research.^3^, ^31^ Third, the chemotherapy regimens all patients received epirubicin/cyclophosphamide followed by taxanes; thus, the overall treatment is relatively consistent.

This study had some limitations. First, it was a retrospective single‐center study with a relatively small sample size and a long time span, which might have led to some bias. Some patients were lost to follow‐up. Second, standardization of the technology for detecting the proportion of Ki‐67‐positive cells using IHC and the representativeness and reproducibility of overall Ki‐67 expression by core needle biopsies before NST remains uncertain. Breast cancer is highly heterogeneous, and we can only evaluate the overall tumor of patients by puncturing part of the tissue. The inability to accurately judge the initial numerical state of Ki‐67_B_ is indeed a limitation of NST. Third, since DFS is only a surrogate endpoint for overall survival, longer follow‐up of non‐pCR patients is crucial to further verify the long‐term clinical benefit and differences between the two methods.

In conclusion, this investigation revealed that the combination of Ki‐67_B_ and Ki‐67_C_ is superior to Ki‐67_T_ in predicting DFS, especially in a longer follow‐up period. Regarding clinical application, this combination could be used as a novel indicator to predict DFS to more clearly identify those patients at high risk and to better determine whether escalation strategies in terms of adjuvant treatment are necessary.

## AUTHOR CONTRIBUTIONS

**Weiwei Zhang:** Conceptualization (equal); data curation (equal); formal analysis (equal); funding acquisition (equal); investigation (equal); methodology (equal); project administration (equal); resources (equal); software (equal); supervision (equal); validation (equal); visualization (equal); writing – original draft (equal); writing – review and editing (equal). **Yinggang Xu:** Conceptualization (equal); data curation (equal); formal analysis (equal); funding acquisition (equal); investigation (equal); methodology (equal); project administration (equal); resources (equal); software (equal); supervision (equal); validation (equal); visualization (equal); writing – original draft (equal); writing – review and editing (equal). **Ye Wang:** Investigation (equal). **Jinzhi He:** Investigation (equal). **Rui Chen:** Investigation (equal). **Xinyu Wan:** Investigation (equal). **Wenjie Shi:** Investigation (equal). **Xiaofeng Huang:** Investigation (equal). **Xiaoqing Shi:** Investigation (equal). **Jue Wang:** Methodology (equal); project administration (equal). **Xiaoming Zha:** Methodology (equal); project administration (equal); supervision (equal); validation (equal); writing – review and editing (equal).

## FUNDING INFORMATION

This research was partially supported by the Jiangsu Province Six Talents Summit Project (WSW‐001), Chinese Society of Clinical Oncology Foundation (Y‐sy2018‐077, Y‐JS2019‐096), National Natural Science Foundation of China (81302305), and Young Scholars Fostering Fund of the First Affiliated Hospital of Nanjing Medical University (PY2021038). Funders had no role in any of the stages from study design to submission of the paper for publication.

## CONFLICT OF INTEREST

The authors have no conflicts of interest to declare.

## ETHICS STATEMENT

The study protocol was conducted in accordance with the Ethical Principles for Medical Research Involving Human Subjects as described in the Declaration of Helsinki (as revised in 2013). This is an observational study. The study protocol was approved by the Ethics and Research Committee of the First Affiliated Hospital of Nanjing Medical University (the ethics number was 2021‐SR‐515).

## PATIENT CONSENT STATEMENT

Informed consent was obtained from each patient (every patient signed an informed consent form before receiving neoadjuvant systemic treatment).

## Supporting information


**Figure S1.** Time‐dependent ROC curve analysis for the percentage change of Ki‐67 at year 3.
ROC, receiver operating characteristic; AUC, area under the curve.

## Data Availability

Data sharing is not applicable to this article as no new data were created or analyzed in this study.

## References

[1] Fisher B , Bryant J , Wolmark N , et al. Effect of preoperative chemotherapy on the outcome of women with operable breast cancer. J Clin Oncol. 1998;16(8):2672‐2685. 9704717 10.1200/JCO.1998.16.8.2672

[2] van der Hage JA , van de Velde CJ , Julien JP , Tubiana‐Hulin M , Vandervelden C , Duchateau L . Preoperative chemotherapy in primary operable breast cancer: results from the European Organization for Research and Treatment of cancer trial 10902. J Clin Oncol. 2001;19(22):4224‐4237. 11709566 10.1200/JCO.2001.19.22.4224

[3] Cortazar P , Zhang L , Untch M , et al. Pathological complete response and long‐term clinical benefit in breast cancer: the CTNeoBC pooled analysis. Lancet. 2014;384(9938):164‐172. 24529560 10.1016/S0140-6736(13)62422-8

[4] Cortazar P , Geyer CE Jr . Pathological complete response in neoadjuvant treatment of breast cancer. Ann Surg Oncol. 2015;22(5):1441‐1446. 25727556 10.1245/s10434-015-4404-8

[5] Houssami N , Macaskill P , von Minckwitz G , Marinovich ML , Mamounas E . Meta‐analysis of the association of breast cancer subtype and pathologic complete response to neoadjuvant chemotherapy. Eur J Cancer 2012;48(18):3342–3354. 22766518 10.1016/j.ejca.2012.05.023

[6] Denkert C , von Minckwitz G , Darb‐Esfahani S , et al. Tumour‐infiltrating lymphocytes and prognosis in different subtypes of breast cancer: a pooled analysis of 3771 patients treated with neoadjuvant therapy. Lancet Oncol. 2018;19(1):40‐50. 29233559 10.1016/S1470-2045(17)30904-X

[7] Symmans WF , Wei C , Gould R , et al. Long‐term prognostic risk after neoadjuvant chemotherapy associated with residual cancer burden and breast cancer subtype. J Clin Oncol. 2017;35(10):1049‐1060. 28135148 10.1200/JCO.2015.63.1010 PMC5455352

[8] Gerdes J , Lemke H , Baisch H , Wacker HH , Schwab U , Stein H . Cell cycle analysis of a cell proliferation‐associated human nuclear antigen defined by the monoclonal antibody Ki‐67. J Immunol. 1984;133(4):1710‐1715. 6206131

[9] de Azambuja E , Cardoso F , de Castro G , Jr., et al. Ki‐67 as prognostic marker in early breast cancer: a meta‐analysis of published studies involving 12,155 patients. Br J Cancer 2007;96(10):1504–1513. 17453008 10.1038/sj.bjc.6603756 PMC2359936

[10] Yerushalmi R , Woods R , Ravdin PM , Hayes MM , Gelmon KA . Ki67 in breast cancer: prognostic and predictive potential. Lancet Oncol. 2010;11(2):174‐183. 20152769 10.1016/S1470-2045(09)70262-1

[11] Li L , Han D , Wang X , et al. Prognostic values of Ki‐67 in neoadjuvant setting for breast cancer: a systematic review and meta‐analysis. Future Oncol (London, England). 2017;13(11):1021‐1034. 10.2217/fon-2016-0428 28088868

[12] Chen X , He C , Han D , et al. The predictive value of Ki‐67 before neoadjuvant chemotherapy for breast cancer: a systematic review and meta‐analysis. Future Oncol (London, England). 2017;13(9):843‐857. 10.2217/fon-2016-0420 28075166

[13] Jain P , Doval DC , Batra U , et al. Ki‐67 labeling index as a predictor of response to neoadjuvant chemotherapy in breast cancer. Jpn J Clin Oncol. 2019;49(4):329‐338. 30753543 10.1093/jjco/hyz012

[14] Denkert C , Loibl S , Müller BM , et al. Ki67 levels as predictive and prognostic parameters in pretherapeutic breast cancer core biopsies: a translational investigation in the neoadjuvant GeparTrio trial. Ann Oncol. 2013;24(11):2786‐2793. 23970015 10.1093/annonc/mdt350

[15] Jones RL , Salter J , A'Hern R , et al. The prognostic significance of Ki67 before and after neoadjuvant chemotherapy in breast cancer. Breast Cancer Res Treat. 2009;116(1):53‐68. 18592370 10.1007/s10549-008-0081-7

[16] Fasching PA , Gass P , Häberle L , et al. Prognostic effect of Ki‐67 in common clinical subgroups of patients with HER2‐negative, hormone receptor‐positive early breast cancer. Breast Cancer Res Treat. 2019;175(3):617‐625. 30868391 10.1007/s10549-019-05198-9

[17] Rossi L , Verrico M , Tomao S , et al. Expression of ER, PgR, HER‐2, and Ki‐67 in core biopsies and in definitive histological specimens in patients with locally advanced breast cancer treated with neoadjuvant chemotherapy. Cancer Chemother Pharmacol. 2020;85(1):105‐111. 31754747 10.1007/s00280-019-03981-5

[18] Miglietta L , Morabito F , Provinciali N , et al. A prognostic model based on combining estrogen receptor expression and Ki‐67 value after neoadjuvant chemotherapy predicts clinical outcome in locally advanced breast cancer: extension and analysis of a previously reported cohort of patients. Eur J Surg Oncol. 2013;39(10):1046‐1052. 23890870 10.1016/j.ejso.2013.06.024

[19] Smith I , Robertson J , Kilburn L , et al. Long‐term outcome and prognostic value of Ki67 after perioperative endocrine therapy in postmenopausal women with hormone‐sensitive early breast cancer (POETIC): an open‐label, multicentre, parallel‐group, randomised, phase 3 trial. Lancet Oncol. 2020;21(11):1443‐1454. 33152284 10.1016/S1470-2045(20)30458-7 PMC7606901

[20] Burstein HJ , Curigliano G , Thürlimann B , et al. Customizing local and systemic therapies for women with early breast cancer: the St. Gallen International Consensus Guidelines for treatment of early breast cancer 2021. Ann Oncol. 2021;32(10):1216‐1235. 34242744 10.1016/j.annonc.2021.06.023 PMC9906308

[21] von Minckwitz G , Huang CS , Mano MS , et al. Trastuzumab emtansine for residual invasive HER2‐positive breast cancer. N Engl J Med. 2019;380(7):617‐628. 30516102 10.1056/NEJMoa1814017

[22] Regan MM , Neven P , Giobbie‐Hurder A , et al. Assessment of letrozole and tamoxifen alone and in sequence for postmenopausal women with steroid hormone receptor‐positive breast cancer: the BIG 1‐98 randomised clinical trial at 8·1 years median follow‐up. Lancet Oncol. 2011;12(12):1101‐1108. 22018631 10.1016/S1470-2045(11)70270-4 PMC3235950

[23] Grizzi G , Ghidini M , Botticelli A , et al. Strategies for increasing the effectiveness of aromatase inhibitors in locally advanced breast cancer: an evidence‐based review on current options. Cancer Manag Res. 2020;12:675‐686. 32099464 10.2147/CMAR.S202965 PMC6996551

[24] Masuda N , Lee SJ , Ohtani S , et al. Adjuvant capecitabine for breast cancer after preoperative chemotherapy. N Engl J Med. 2017;376(22):2147‐2159. 28564564 10.1056/NEJMoa1612645

[25] Harbeck N , Rastogi P , Martin M , et al. Adjuvant abemaciclib combined with endocrine therapy for high‐risk early breast cancer: updated efficacy and Ki‐67 analysis from the monarchE study. Ann Oncol. 2021;32(12):1571‐1581. 34656740 10.1016/j.annonc.2021.09.015

[26] Modi S , Saura C , Yamashita T , et al. Trastuzumab deruxtecan in previously treated HER2‐positive breast cancer. N Engl J Med. 2020;382(7):610‐621. 31825192 10.1056/NEJMoa1914510 PMC7458671

[27] Petrelli F , Viale G , Cabiddu M , Barni S . Prognostic value of different cut‐off levels of Ki‐67 in breast cancer: a systematic review and meta‐analysis of 64,196 patients. Breast Cancer Res Treat. 2015;153(3):477‐491. 26341751 10.1007/s10549-015-3559-0

[28] Pistelli M , Merloni F , Crocetti S , et al. Prognostic impact of Ki‐67 change in locally advanced and early breast cancer after neoadjuvant chemotherapy: a single institution experience. J Oncol. 2021;2021:5548252‐5548257. 34054952 10.1155/2021/5548252 PMC8112947

[29] Matsubara N , Mukai H , Masumoto M , et al. Survival outcome and reduction rate of Ki‐67 between pre‐ and post‐neoadjuvant chemotherapy in breast cancer patients with non‐pCR. Breast Cancer Res Treat. 2014;147(1):95‐102. 25106660 10.1007/s10549-014-3084-6

[30] Tan S , Fu X , Xu S , et al. Quantification of Ki67 change as a valid prognostic indicator of luminal B type breast cancer after neoadjuvant therapy. Pathol Oncol Res. 2021;27:1609972. 34987312 10.3389/pore.2021.1609972 PMC8722379

[31] Untch M , Konecny GE , Paepke S , von Minckwitz G . Current and future role of neoadjuvant therapy for breast cancer. Breast. 2014;23(5):526‐537. 25034931 10.1016/j.breast.2014.06.004

