# Supplementary figures and images for: Prognostic analysis of three forms of Ki‐67 in patients with breast cancer with non‐pathological complete response before and after neoadjuvant systemic treatment
Source: Cancer Med. 2023 Feb 16;12(8):9363–72. doi: 10.1002/cam4.5693 (PMC10166904; doi:10.1002/cam4.5693)

ROC Curve at time = 36 months

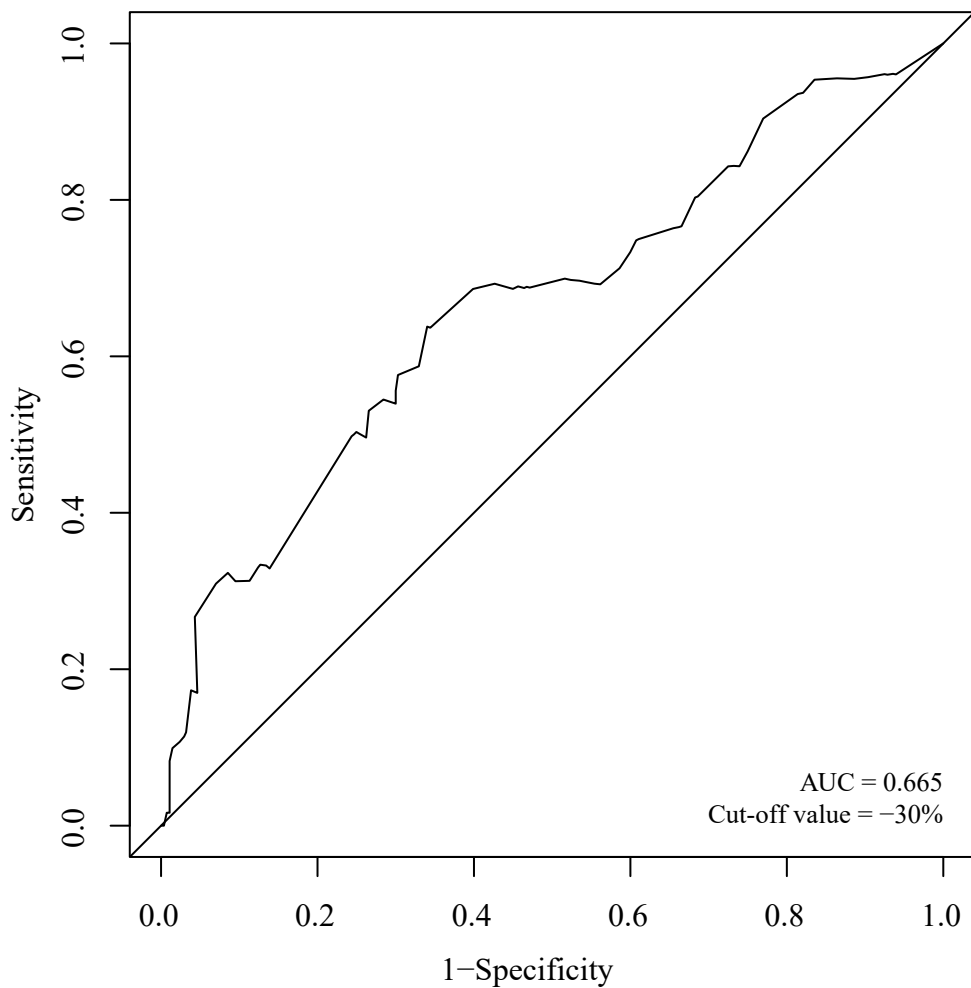

Supplement: Supplementary file 1 — Figure S1. Time‐dependent ROC curve analysis for the percentage change of Ki‐67 at year 3. ROC, receiver operating characteristic; AUC, area under the curve. [file CAM4-12-9363-s001.pdf]
